# New Data on *Rhinogobius chiengmaiensis* and *Rhinogobius mekongianus* in Thailand by DNA Barcoding and Morphological Methods

**DOI:** 10.3390/ani15060871

**Published:** 2025-03-19

**Authors:** Siriluck Tuncharoen, Paiboon Panase, Nontree Panprommin, Eakapol Wangkahart, Supranee Ruenkoed, Keatipong Mongkolwit, Dutrudi Panprommin

**Affiliations:** 1 School of Agriculture and Natural Resources, University of Phayao, Phayao 56000, Thailand; plagutt@yahoo.com (S.T.); tong33_panamagigas@hotmail.com (P.P.); 2 Department of Fisheries, Aquatic Plants and Ornamental Fish Research Institute, Bangkok 10900, Thailand; nontreep@hotmail.com; 3 Faculty of Technology, Mahasarakham University, Mahasarakham 44150, Thailand; eakapol.w@msu.ac.th; 4 Advance Pharma Vietnam Co., Ltd., Ho Chi Minh City 10000, Vietnam; supranee.rue@ahb.com.vn (S.R.); keatipong.poo@ahb.com.vn (K.M.)

**Keywords:** morphological identification, DNA barcoding, cytochrome c oxidase I, species identification, *Rhinogobius*, Oxudercidae

## Abstract

A combination of morphological analysis and DNA barcoding (partial sequences of the cytochrome c oxidase I (COI) gene) was used to differentiate four gobiid fish species in the family Oxudercidae. *Rhinogobius chiengmaiensis* and *Rhinogobius mekongianus* were found in Thailand, while *Eugnathogobius siamensis* and *Pseudogobiopsis oligactis* were used for comparative purposes. Morphological identification relied on appearances, counts, and measurements. The 707-base pair COI sequences from eleven samples of four gobiid species were compared with reference sequences in public databases to confirm their scientific names. The average AT content was 51.8 ± 0.5% and the GC content was 48.2 ± 0.5%. Intraspecific genetic distances ranged from 0.00–0.28%, while interspecific genetic distances ranged from 0.86–16.63%. A neighbor-joining (NJ) phylogenetic tree depicted the relationships among the COI sequences of these species. Morphological analysis and COI sequences successfully distinguished the four gobiid species. Notably, the COI sequences of *R. chiengmaiensis*, *R. mekongianus*, and *E. siamensis* were previously unreported, hence, this study is the first report to add their sequences to public databases. These results can serve as valuable information for the management of aquatic resources, conservation, and aquaculture efforts.

## Simple Summary

Morphology and DNA barcoding were utilized for the identification of two *Rhinogobius* species, *R. chiengmaiensis* and *R. mekongianus*, along with *Eugnathogobius siamensis* and *Pseudogobiopsis oligactis* for comparative analysis. Eleven samples were collected from natural habitats in Thailand. The collaboration between morphological analysis and DNA barcoding reveals the accuracy and precision in distinguishing these four gobiid species. Our research provides essential reference data to support the management of fishery resources, aquaculture practices, and conservation efforts.

## 1. Introduction

The family Oxudercidae (or Gobionellidae) belongs to the order Gobiiformes, and comprises approximately 86 genera and 598 species that are found worldwide from freshwater to marine habitats [1]. Species in this family were previously classified in the subfamily Oxudercinae of the family Gobiidae according to their taxonomic arrangement, following Nelson [2]. Later, when the taxonomy of fish in this taxa was verified, it was revised that the subfamily Oxudercinae was separated from the family Gobiidae with the status of the family Oxudercidae [1]. One distinguishing feature of the family Oxudercidae, as compared to the family Gobiidae, is the presence of elongated and slender suspensoria structures [1,3].

*Rhinogobius*, a genus within the family Oxudercidae, contains the most species of freshwater gobies found in the lakes and streams across East and Southeast Asia, including Thailand [4,5,6]. *Rhinogobius* species exhibit characteristics that are commonly observed in gobies, such as their small body size, the fusion of their pelvic fins to form a disc-like structure on the ventral side, and an elongated and rounded body [7,8]. Moreover, this genus exhibits an elongated form of the head, characterized by a long snout. Presently, the genus *Rhinogobius* is recognized to encompass more than 80 distinct species, and ongoing taxonomic research is consistently discovering and documenting new species such as *R. sangenloensis* in Southern China [5], *R. maxillivirgatus* in Eastern China [9], *R. yangminshanensis* in Taiwan [10], and *R. aonumai aonumai* and *R. aonumai ishigakiensis* in Japan [11]. In addition, two new species, *R. rong* and *R. nami*, have recently been described from central Vietnam [12]. However, Panitvong [13] notes that only three *Rhinogobius* species are currently recognized in Thailand: *R. chiengmaiensis*, *R. mekongianus*, and *R. giurinus*.

*Rhinogobius chiengmaiensis* exhibits a broad distribution in the upper region of the Chao Phraya river, whereas *R. mekongianus* is found inhabiting the Mekong river basin [14]. In 2013, Panitvong [13] found *R. giurinus* within a stream located in Chiang Rai province, Northern Thailand. Nevertheless, there remains limited documentation of the presence of this species in Thailand. Therefore, this study focused on two species, *R. chiengmaiensis*, and *R. mekongianus*. Although these two species are found in different natural habitats, they share some similarities in appearance, such as small size, elongated body shape, and the pattern of colors. They are currently becoming popular as ornamental fish, causing the natural population trend to decrease, especially in the case of *R. chiengmaiensis*. The conservation statuses of *R. chiengmaiensis* and *R. mekongianus* were assessed by the International Union for Conservation of Nature (IUCN) as vulnerable (VU) and least concern (LC), respectively (as of February 2025). Hence, both morphological and molecular identification methods were required to distinguish between these two species.

The morphological identification of fish species involves the assessment of meristic and morphometric characteristics. Meanwhile, morphological identification, DNA barcoding, or the standard sequences of the cytochrome c oxidase I (COI) gene have been commonly employed for global species bioidentification [15], including the case of fishes [16,17,18,19,20]. Both morphological identification and DNA barcoding have distinct advantages and disadvantages. Morphological identification is typically conducted by experienced fish taxonomists, while DNA barcoding is employed for identifying fish species even in cases of incomplete samples such as fish fillets [21], or the various developmental stages such as the eggs [22] or larval stage [23]. Collaboration between morphological identification and DNA barcoding enhances the accuracy of fish species identification and contributes to the expansion of the COI gene sequences in public databases for further analysis.

The objective of this research was to differentiate between two species of the genus *Rhinogobius*, namely *R. chiengmaiensis* and *R. mekongianus*, through the application of both morphological identification and DNA barcoding techniques. Furthermore, the research also focused on studying two other species within the family Oxudercidae, namely *Eugnathogobius siamensis* and *Pseudogobiopsis oligactis*. These two species were analyzed in comparison to two species of the genus *Rhinogobius*. This research provided the first report of the COI sequences of *R. chiengmaiensis*, *R. mekongianus*, and *E. siamensis*. The COI sequences of all four species were expanded and deposited into the GenBank and BOLD databases to serve as reference sequences for aiding in the identification of unknown species and establishing a database for specifying of new species within the family Oxudercidae, especially the genus *Rhinogobius*.

## 2. Materials and Methods

### 2.1. Sample Collection

**Figure 1:**
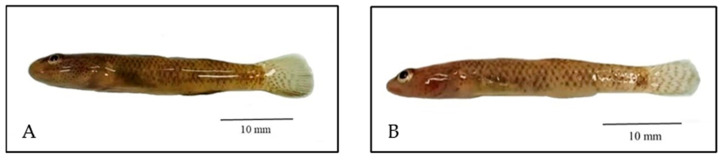
*Rhinogobius chiengmaiensis* (**A**) and *Rhinogobius mekongianus* (**B**).

Seven live samples of two species belonging to the genus *Rhinogobius*, *R. chiengmaiensis* and *R. mekongianus* were obtained using swings in September 2024 from collectors of fish from nature with known sources (Figure 1 and Table 1). In addition, four samples of two other species of the family Oxudercidae, *E. siamensis* and *P. oligactis*, were also received. All samples were photographed and anesthetized with 0.2 g/L of MS-222 (Sigma, St. Louis, MO, USA) solution. Whole samples were preserved in absolute ethanol at room temperature for morphological identification. This animal use protocol has been approved by the Institutional Animal Care and Use Committee, University of Phayao (No. UP-AE 1-011-67).

### 2.2. Morphological Identification

After 2–3 min of immersion in ethanol, all samples were immediately identified based on morphological appearances, including body shape, mouth and eye size. Counts and measurements following the Nagao Natural Environment Foundation’s criteria [24] were performed on the left side of the samples. Fin rays were counted for the dorsal, pectoral, pelvic, and anal fins. The longitudinal, predorsal, and circumcaudal scales were also recorded. Measurements were taken point to point with digital vernier calipers to the nearest 0.01 mm. Subunits of the head were presented as proportions of head length (HL). Measurements of head and body parts were provided as proportions of standard length (SL).

### 2.3. Molecular Identification

After morphological identification, a small piece of right pectoral fin from each sample was used for the extraction of the genomic DNA using the GF-1 Nucleic Acid Extraction kit (Vivantis Technologies Sdn. Bhd., Selangor Darul Ehsan, MA, USA). The quantity and quality of the extracted DNA was determined using a NanoDrop Microvolume Spectrophotometer (Thermo Fisher Scientific Inc., Waltham, MA, USA) and 1.2% agarose gel electrophoresis at 100 V for 30 min, respectively.

A 707 bp fragment of the COI gene was amplified for each sample using the equal volume of each 10 µM primer, namely FishF1 (5′-TCAACCAACCACAAAGACATTGGCAC-3′), FishF2 (5′-TCGACTAATCATAAAGATATCGGCAC-3′), FishR1 (5′-TAGACTTCTGGGTGGCCAAAGAATCA-3′), and FishR2 (5′-ACTTCAGGGTGACCGAAGAATCAGAA-3′) [20], with the PCR technique. Each PCR reaction mixture included of 1 µL of extracted DNA (approximately 100 ng/µL), 2.25 µL of 10× *Taq* buffer, 0.8 µL of 50 mM MgCl_2_, 1 µL of 2.5 mM dNTP mix, 1 µL of 10 µM mixed primers, 0.2 µL of 1 U *Taq* DNA polymerase (BIO-HELIX Co., Ltd., New Taipei City, Taiwan), and nuclease-free water to 25 µL. The thermal cycling conditions included an initial denaturation at 95 °C for 2 min followed by 35 cycles of denaturation at 94 °C for 30 s, annealing at 54 °C for 30 s, and extension at 72 °C for 1 min, with a final extension at 72 °C for 10 min. The PCR products were analyzed using 1.2% agarose gel electrophoresis stained with Novel Juice (BIO-HELIX Co., Ltd., New Taipei City, Taiwan). The target bands were then purified to remove unwanted reagents using the HiYield™ Gel/PCR DNA Fragments Extraction kit (RBC Bioscience Corp., New Taipei City, Taiwan), following the manufacturer’s instructions.

The purified PCR products were sequenced bidirectionally at ATGC Co., Ltd. (Pathum Thani, Thailand) using primer FishF1/FishF2 for the 3′ end and FishR1/FishR2 for the 5′ end. Two sequences of each sample were aligned and assembled using the Clustal Omega program [25] version 1.2.4. All sequences covering the primer sequences were searched for the stop codon, deletion, and insertion using the Open Reading Frame Finder website (ORF finder; https://www.ncbi.nlm.nih.gov/orffinder/, accessed on 12 October 2024). The scientific name of each sample was investigated by comparing its sequence with reference sequences in two public databases, namely the GenBank (https://www.ncbi.nlm.nih.gov/, accessed on 12 October 2024) using the Basic Local Alignment Search Tool (BLAST) program [26] and BOLD (https://boldsystems.org/, accessed on 12 October 2024). Each sample was assigned a scientific name based on the top match with a minimum of 99% sequence similarity.

The percentage of base composition of the four gobiid species was calculated using the MEGA11 program [27]. The intraspecific and interspecific genetic distances were calculated using the MEGA11 program with the Kimura-2-parameter (K2P) distance model [28]. Because the COI sequences of *R. chiengmaiensis*, *R. mekongianus*, and *E. siamensis* have not been reported in any databases, several sequences of other fish species of the genus *Rhinogobius* and *P. oligactis* were downloaded from GenBank and BOLD databases for construction of a neighbor-joining (NJ) tree [29] to analyze their evolutionary relationship. The tree was generated using the MEGA11 program with 1000 bootstrap replications, using nucleotide sequences of equal length. A COI sequence from *Oxyeleotris marmorata*, a member of the family Butidae, was used as an outgroup.

## 3. Results

### 3.1. Morphological Identification

Although these two species of the genus *Rhinogobius* showed very similar characteristics, their appearances were clearly different (Table 2), including two other members of the family Oxudercidae, *E. siamensis* and *P. oligactis*. A short description of the four gobiid species examined in this study is provided below.

*Rhinogobius chiengmaiensis* Fowler, 1934

D1. vi–vii; D2. i,7–8; P1. i,14; P2. 12 (total); A. i,6–7; C. branched 10

First dorsal fin with 6–7 single rays; i,7-8 second dorsal fin: i,14 pectoral fin rays: 12 (total) pelvic fin rays: i,6-7 anal fin rays: 10 caudal branched rays; ctenoid scale; longitudinal scales 29–30; predorsal scales 3–4; circumcaudal scales 12; body elongate, moderate slender (15.85 ± 0.31%SL); pelvic fins origin slightly in front of opercula margin (29.71 ± 2.56 vs. 31.82 ± 1.67%SL); pelvic fins length 19.37 ± 1.15%SL; mouth moderate large (35.50 ± 3.86%HL), maxillary extending to middle of eyes; eyes diameter 20.16 ± 1.39%HL.

*Rhinogobius mekongianus* (Pellegrin & Fang, 1940)

D1. vi; D2. i,7-8; P1. i,14; P2. 12 (total); A. i,6-7; C. branched 10

First dorsal fin with 6–7 single rays; i,7-8 second dorsal fin: i,14 pectoral fin rays: 10 (total) pelvic fin rays: i,6 anal fin rays: 12 caudal branched rays; ctenoid scale; longitudinal scales 29–30; predorsal scales 3–4; circumcaudal scales 12; body elongate, very slender (13.28 ± 0.54%SL); pelvic fins origin slightly in front of opercula margin (29.33 ± 0.18 vs. 30.50 ± 0.38%SL); pelvic fins length 17.88 ± 1.17%SL; mouth moderate large (39.19 ± 2.06%HL), maxillary extending to middle of eyes; eyes diameter 19.74 ± 0.57%HL.

*Eugnathogobius siamensis* (Fowler, 1934)

D1. vi; D2. i,6-7; P1. i,16-18; P2. 10 (total); A. i,6; C. branched 12-14

First dorsal fin with 6 single rays; i,6-7 second dorsal fin: i,16-18 pectoral fin rays: 10 (total) pelvic fin rays: i,6 anal fin rays: 12-14 caudal branched rays; large ctenoid scale; longitudinal scales 24; predorsal scales 7; circumcaudal scales 12; body elongate, moderate slender (18.08 ± 2.29%SL); pelvic fins origin slightly behind opercula margin (33.31 ± 1.54 vs. 32.60 ± 1.24%SL); pelvic fins length 24.85 ± 0.35%SL; caudal fin length 22.61 ± 3.17%SL; mouth large (48.33 ± 12.41%HL), maxillary extending well beyond posterior margin of eyes in male and extending to middle of eyes in female; small eyes, diameter 13.16 ± 0.72%HL.

*Pseudogobiopsis oligactis* (Bleeker, 1875)

D1. vi; D2. i,6-7; P1. i,14; P2. 12-14 (total); A. i,7-8; C. branched 12-14

First dorsal fin with 6 single rays; i,6-7 second dorsal fin: i,14 pectoral fin rays: 12-14 (total) pelvic fin rays: i,7-8 anal fin rays: 12-14 caudal branched rays; large ctenoid scale; longitudinal scales 24–25; predorsal scales 7; circumcaudal scales 10; body elongate, moderate slender (15.88 ± 0.51%SL); pelvic fins origin slightly behind opercula margin (36.06 ± 0.37 vs. 35.86 ± 3.28%SL); pelvic fins length 22.75 ± 0.36%SL; mouth large (63.24 ± 0.80%HL), maxillary extending well beyond posterior margin of eyes; small eyes, diameter 12.63 ± 0.19%HL. The diagnostic key to all four gobiid species in this study was provided as follows:

Diagnostic key to species of four gobiid in this study
1aLarge ctenoid scales; longitudinal scales 24–25; predorsal scales 7; pelvic fins origin slightly behind the opercular margin21bModerate ctenoid scales; longitudinal scales 29–30; predorsal scales 3–4; pelvic fins origin slightly behind the opercular margin 32aLarge head, head length 35.86 ± 3.28%SL, head width 62.30 ± 3.15%HL; large mouth (63.24 ± 0.80%HL), maxillary extending well beyond the posterior margin of the eyes; pelvic fin length 22.75 ± 0.36%SL*Pseudogobiopsis oligactis*2bSmall head, head length 32.60 ± 1.24%SL, head width 55.61 ± 3.85%HL; moderately large mouth (48.33 ± 12.41%HL), maxillary extending from the middle of the eyes to well beyond the posterior margin of the eyes; pelvic fin length 24.85 ± 0.35%SL *Eugnathogobius siamensis*3aModerately large mouth, maxillary extending to the middle of the eyes (35.50 ± 3.86%HL); moderately slender body, body depth at pelvic fin origin 15.85 ± 0.31%SL; pelvic fin length 19.37 ± 1.15%SL; caudal fin length 24.77 ± 0.54%SL *Rhinogobius chiengmaiensis*3bModerately large mouth, maxillary extending to the middle of the eyes (39.19 ± 2.06%HL); very slender body, body depth at pelvic fin origin 13.28 ± 0.54%SL; pelvic fin length 17.88 ± 1.17%SL; caudal fin length 27.34 ± 2.28%SL *Rhinogobius mekongianus*

### 3.2. Molecular Identification

The 707 bp fragments of the COI gene were successfully amplified from all samples using the PCR technique. The 235 amino acid residues were translated without stop codon, deletion, or insertion for any sequences. From sequence alignment, two sequences of *R. chiengmaiensis* showed 100% similarity (1 haplotype). While five sequences of *R. mekongianus* showed 99.4% similarity (4 haplotypes), four bases were different. Two COI sequences of *R. chiengmaiensis* and five COI sequences of *R. mekongianus* were compared to reference sequences in GenBank, showing the highest similarity to *Rhinogobius virgigena* at 95.85% and 95.44–95.72%, respectively, with 99% query coverage. Similarly, these sequences exhibited the highest similarity to *R. virgigena* in the BOLD database, with an identity of 96.22% for *R. chiengmaiensis* and 95.91–96.22% for *R. mekongianus* (Table 3).

The COI sequences of *E. siamensis* and *P. oligactis* demonstrated complete similarity, with each species showing a haplotype. Sixteen bases were different from each species. For comparison with the reference sequences in the GenBank, two COI sequences of *E. siamensis* showed the highest similarity to *P. oligactis* at 98.14% with 99% query coverage. Similarly, two COI sequences of *P. oligactis* showed the highest similarity to *P. oligactis* at 99.29% with 100% query coverage. Based on the BOLD database, two sequences of each species showed the highest similarity to *P. oligactis* at 98.93% and 99.85% identities, respectively (Table 3).

The average base compositions of eleven COI sequences were T (29.0 ± 1.3%), C (28.6 ± 0.1%), A (22.9 ± 0.9%), and G (19.5 ± 0.5%), as shown in Table 4. The GC content was 48.2 ± 0.5% at all sites, while the AT content was 51.8 ± 0.5%. *Pseudogobiopsis oligactis* presented the highest GC content (49.1 ± 0.0%), while *R. mekongianus* had the lowest (47.8 ± 0.1%).

The intraspecific genetic distances were 0.00% for *R. chiengmaiensis*, *E. siamensis* and *P. oligactis*, and 0.28% for *R. mekongianus* (Table 5), while the average intraspecific distance was 0.07%. In contrast, the interspecific genetic distances ranged from 0.86–16.63%. The lowest distance was between *R. chiengmaiensis* and *R. mekongianus* (0.86%). The highest distances were between *R. chiengmaiensis* and *P. oligactis* (16.63%) and *R. chiengmaiensis* and *E. siamensis* (15.38%), followed by *R. mekongianus* and *P. oligactis* (12.00%) and *R. mekongianus* and *E. siamensis* (11.02%), respectively. Meanwhile, the distance between *P. oligactis* and *E. siamensis* was 1.64%. The average interspecific distance was 9.59%. Thus, the interspecific genetic distance was 137-fold greater than the intraspecific genetic distance.

**Figure 2:**
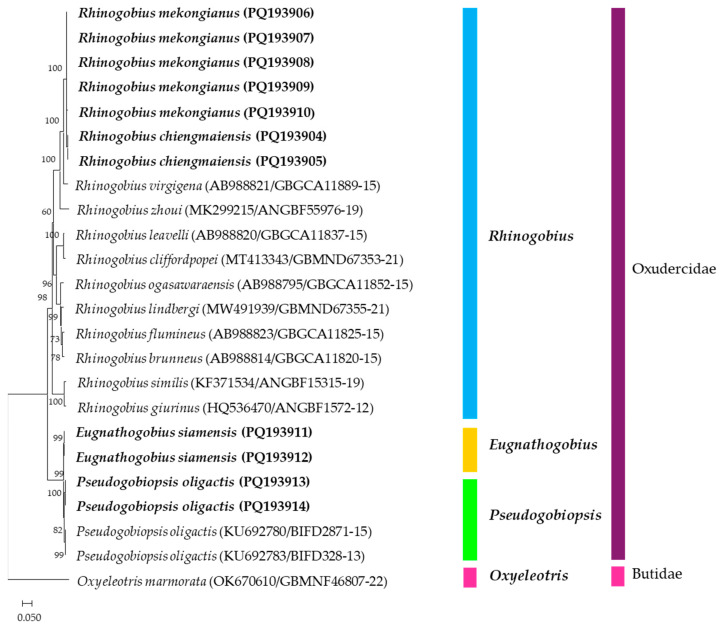
A neighbor-joining (NJ) tree was constructed using eleven COI nucleotide sequences from four gobiid species, highlighted in bold, in comparison with multiple sequences sourced from the GenBank and BOLD databases (GenBank and BOLD accession numbers). The bootstrap values are presented above the branches. A COI sequence of *Oxyeleotris marmorata* was designated as the outgroup species. The scale bar corresponds to the number of substitutions per site.

The NJ phylogenetic tree presented the relationship between the COI sequences of the four gobiid species of this study and other species retrieved from the GenBank and BOLD databases (Figure 2). Two major clades were clearly delineated, one comprising *Rhinogobius* species and the other including *E. siamensis* and *P. oligactis*, family Oxudercidae. Within these groups, *R. chiengmaiensis* showed the greatest similarity to *R. mekongianus*, while *E. siamensis* and *P. oligactis* clustered together. Additionally, the COI sequence of *O. marmorata*, family Butidae, was distinctly separated from the other sequences.

## 4. Discussion

The morphometric characters of *R. chiengmaiensis* and *R. mekongianus* provided in this study aligned with findings from previous research [4,14,30,31]. *Rhinogobius chiengmaiensis* and *R. mekongianus* exhibited quite similar appearances; however, they differed in certain characteristics such as the number of rays in the pelvic fin, anal fin, and caudal branch, as well as the diameter of the eyes. Additionally, a notable distinguishing characteristic was the body shape, with *R. mekongianus* being more slender compared to *R. chiengmaiensis*.

The concise characterizations of *E. siamensis* and *P. oligactis* presented in this study were also consistent with the descriptions provided in previous research [32,33]. Considerable confusion has arisen in accurately distinguishing between these two gobiid fish species due to their similar appearances, particular the coloration of their head and body [24] and greatly enlarged jaws [34]. In addition, these two species have been greatly confused, as evidenced by their synonyms being swapped between genera in FishBase, a global fish species database (https://www.fishbase.se/, accessed on 31 October 2024). At one time, the invalid names *Pseudogobiopsis siamensis* and *Eugnathogobius oligactis* were used for *E. siamensis* and *P. oligactis*, respectively. In 2009, Larson [32] conducted a review of the gobiid fish genera *Eugnathogobius* and *Pseudogobiopsis*, which significantly enhanced the ability to distinguish between these two species.

To accurately identify these four gobiid species, which exhibit highly similar external characteristics, it is essential to employ molecular data and DNA barcoding, alongside traditional taxonomic methods. This integrative approach ensures precise identification of these closely related fish species. Xia et al. [9] examined the COI sequences of the newly discovered species *R. maxillivirgatus*, revealing that it is closely related to its nearest species, *R. wuyanlingensis*, while still being distinct. In addition, the 707 bp fragments of the COI gene, which include primer sequences in this study, did not compromise comparability with previous studies that utilized a 655 bp fragment, as the same primers were employed for fish species identification.

Due to low identity percentages, the COI sequences of two fish species belonging to the genus *Rhinogobius* in Thailand have not yet been reported in any databases. Thus, this study was the first investigation to report the COI sequences of *R. chiengmaiensis* and *R. mekongianus*. Additionally, the COI sequences of *E. siamensis* have also been initially provided. A search in the Data Portal (https://portal.boldsystems.org/, accessed on 26 February 2025) of the BOLD database for these three fish species also confirmed that no records had been reported previously. A total of eleven COI sequences were deposited in the GenBank database with accession numbers PQ193904-PQ193914 (Table 1).

The average AT content (51.8 ± 0.5%) was greater than the average GC content (48.2 ± 0.5%) due to the average T being the highest in the base composition of eleven sequences, followed by C and A, respectively. The highest average thymine base composition was exhibited in several fishes including freshwater fishes of Bangladesh [16], marine and coastal fishes of Bangladesh [17], fish species in the Taiwan Strait [18], four fish species in the family Notopteridae [19], and *Wallago attu* [35]. Furthermore, the average G content was the lowest, representing a clear pattern of anti-G bias [16,17].

This study confirmed that the COI sequences were effective for distinguishing the four gobiid species based on genetic distance analysis. The average intraspecific genetic distances were low (0.00–0.28%), while the average interspecific genetic distances were significantly higher (0.86–16.63%). Generally, the intraspecific genetic distances based on the COI gene for animal species are usually less than 2% [36], including fishes [20]. These findings aligned with previous studies on fish species in the Taiwan Strait [18], four fish species in the family Notopteridae [19], and Cyprinidae fish in the midstream of the Yangtze river [37]. The average interspecific genetic distance was 137 times higher than the average intraspecific genetic distance. Hebert et al. [38] suggested that a 10-fold COI sequence difference between the average interspecific and intraspecific differences serve as a criterion for animal species differences. However, the small sample size in this study was one of its limitations.

The evolutionary relationship of the COI sequences among the four fish species and the other species is shown in Figure 2, according to the fish taxonomy of Nelson et al. [1]. The species in the family Oxudercidae, which consisted of *Rhinogobius* spp., *E. siamensis*, and *P. oligactis*, were clearly separated from *O. marmorata*, that belongs to the family Butidae. One major clade was the *Rhinogobius* species. The COI sequences of *R. chiengmaiensis* and *R. mekongianus* were the closest species, which agreed with their morphological identification, followed by *R. virgigena* and other *Rhinogobius* species. The second clade comprised *E. siamensis* and *P. oligactis*, indicating a close evolutionary relationship between these two species. The genera *Eugnathogobius* and *Pseudogobiopsis* were categorized as a part of the Mugilogobius lineage, whereas the genus *Rhinogobius* was classified to the Acanthogobius lineage, for which the phylogenetic analysis was similar to studies conducted by Agorreta et al. [39].

At present, these four gobiid species are increasingly popular as ornamental fish. They are often collected from the wild, which may lead to a decline in their natural populations. Furthermore, the IUCN has assessed one species, *R. chiengmaiensis*, as vulnerable (VU), indicating that this species is at considerable risk of becoming endangered in its natural habitat unless effective conservation measures are implemented [40]. The remaining species were assessed as least concern (LC). Therefore, promoting the commercial breeding of these fish can resolve this problem. A few research studies have been conducted on the breeding of these four gobiid fish species, including *R. chiengmaiensis* [41]. However, these fish have very similar appearances, making accurate species identification necessary before breeding. Furthermore, the results of this study will contribute to species identification and serve as information for future research on new fish species in the genus *Rhinogobius*.

## 5. Conclusions

The species *R. chiengmaiensis* and *R. mekongianus* have been identified through both morphological and molecular methods. Additionally, *E. siamensis* and *P. oligactis*, members of the family Oxudercidae, were also identified. The morphology of the four gobiid species exhibited distinctive characteristics, although some species displayed considerable similarity. For molecular identification, DNA barcoding proves to be an effective approach for distinguishing between these four species, considering both intraspecific and interspecific genetic distances as well as phylogenetic analysis. Moreover, this study is the first to report the COI sequences for *R. chiengmaiensis*, *R. mekongianus*, and *E. siamensis*. These findings can enhance selective breeding by providing molecular marker for species identification and diversity assessment. Additionally, the COI sequences of the four gobiid species serve as essential references for identifying new gobiid species.

## Figures and Tables

**Table 1:** Primary information about eleven COI sequences in this study.

Species	GenBank Accession No.	Sample Size	Collection Site
*Rhinogobius chiengmaiensis*	PQ193904-PQ193905	2	Ping river basin, Chiang Mai province
*Rhinogobius mekongianus*	PQ193906-PQ193910	5	Kok River, Chiang Mai province
*Eugnathogobius siamensis*	PQ193911-PQ193912	2	Surat Thani province
*Pseudogobiopsis oligactis*	PQ193913-PQ193914	2	Satun province

**Table 2:** Morphometry of *R. chiengmaiensis*, *R. mekongianus*, *E. siamensis*, and *P. oligactis*.

Characters	*R. chiengmaiensis*	*R. mekongianus*	*E. siamensis*	*P. oligactis*
SL (mm)	28.82 ± 0.51	29.16 ± 2.95	30.48 ± 2.98	29.42 ± 3.68
As % of SL				
Head length	31.82 ± 1.67	30.50 ± 0.38	32.60 ± 1.24	35.86 ± 3.28
Head width	16.46 ± 3.06	15.23 ± 1.90	18.11 ± 0.57	22.39 ± 3.17
Body depth at P2	15.85 ± 0.31	13.28 ± 0.54	18.08 ± 2.29	15.88 ± 0.51
Body depth at A	14.80 ± 0.24	11.78 ± 0.37	16.49 ± 1.94	13.52 ± 0.72
Snout to D1	37.28 ± 1.59	39.66 ± 2.02	39.56 ± 3.06	45.14 ± 1.75
Snout to D2	59.50 ± 2.57	59.01 ± 3.47	61.91 ± 2.21	58.13 ± 0.89
Snout to A	65.08 ± 1.89	64.27 ± 0.31	65.00 ± 1.75	63.29 ± 2.38
Snout to P2	29.71 ± 2.56	29.33 ± 0.18	33.31 ± 1.54	36.06 ± 0.37
D1 base length	15.24 ± 2.40	14.46 ± 1.18	13.16 ± 0.13	8.84 ± 0.50
D1 base length	20.62 ± 2.94	17.39 ± 0.98	16.95 ± 0.29	16.31 ± 1.47
A base length	14.19 ± 0.01	13.72 ± 0.17	15.85 ± 1.16	13.31 ± 3.12
C length	24.77 ± 0.54	27.34 ± 2.28	22.61 ± 3.17	-
Caudal peduncle length	20.60 ± 1.03	19.72 ± 0.15	20.53 ± 1.52	13.95 ± 2.88
Caudal peduncle depth	11.55 ± 0.38	9.62 ± 0.54	11.38 ± 0.44	10.56 ± 0.56
P1 length	20.74 ± 6.94	25.48 ± 1.84	28.63 ± 0.56	22.70 ± 0.37
P2 length	19.37 ± 1.15	17.88 ± 1.17	24.85 ± 0.35	22.75 ± 0.36
As % of HL				
Snout length	29.76 ± 1.44	27.92 ± 2.71	27.98 ± 0.75	27.79 ± 0.01
Eye diameter	20.16 ± 1.39	19.74 ± 0.57	13.16 ± 0.72	12.63 ± 0.19
Postorbital length	50.08 ± 0.04	52.34 ± 2.13	58.87 ± 0.03	59.58 ± 0.17
Interorbital space	21.93 ± 0.01	16.35 ± 1.22	19.13 ± 0.03	19.99 ± 1.23
snout to maxilla	35.50 ± 3.86	39.19 ± 2.06	48.33 ± 12.41	63.24 ± 0.80
Head width	51.54 ± 6.93	49.97 ± 6.87	55.61 ± 3.85	62.30 ± 3.15

SL: standard length; HL: head length; D1: first dorsal fin; D2: second dorsal fin; P1: pectoral fin; P2: pelvic fin; A: anal fin; C: caudal fin. The values are presented as the mean ± standard error.

**Table 3:** Comparison of the COI sequences of four gobiid species with reference sequences in the GenBank and BOLD databases, percentage identity and query cover.

Species	GenBank Accession No.	GenBank			BOLD	
		Species	% Identity	Query Cover (%)	Species	% Identity
*R. chiengmaiensis*	PQ193904- PQ193905	*Rhinogobius virgigena*	95.85	99	*Rhinogobius virgigena*	96.22
*R. mekongianus*	PQ193906- PQ193910	*Rhinogobius virgigena*	95.44–95.72	99	*Rhinogobius virgigena*	95.91–96.22
*E. siamensis*	PQ193911- PQ193912	*Pseudogobiopsis oligactis*	98.14	99	*Pseudogobiopsis oligactis*	98.93
*P. oligactis*	PQ193913- PQ193914	*Pseudogobiopsis oligactis*	99.29	100	*Pseudogobiopsis oligactis*	99.85

**Table 4:** The average nucleotide composition, GC content, and AT content (%) of all sites in the COI sequences of the four fish species.

Species	Nucleotide Composition (%)				%GC Content	%AT Content
	**T**	**C**	**A**	**G**		
*R. chiengmaiensis*	29.7 ± 0.0	28.8 ± 0.0	22.3 ± 0.0	19.2 ± 0.0	48.0 ± 0.0	52.0 ± 0.0
*R. mekongianus*	30.0 ± 0.1	28.5 ± 0.0	22.2 ± 0.1	19.3 ± 0.1	47.8 ± 0.1	52.2 ± 0.1
*E. siamensis*	27.4 ± 0.0	28.6 ± 0.0	24.3 ± 0.0	19.7 ± 0.0	48.3 ± 0.0	51.7 ± 0.0
*P. oligactis*	27.3 ± 0.0	28.7 ± 0.0	23.6 ± 0.0	20.4 ± 0.0	49.1 ± 0.0	50.9 ± 0.0
Average	29.0 ± 1.3	28.6 ± 0.1	22.9 ± 0.9	19.5 ± 0.5	48.2 ± 0.5	51.8 ± 0.5

The values are presented as the mean ± standard error.

**Table 5:** The average K2P intraspecific and interspecific genetic distances (%) were determined for the four fish species.

Species	*R. chiengmaiensis*	*R. mekongianus*	*E. siamensis*	*P. oligactis*
*R. chiengmaiensis*	0.00			
*R. mekongianus*	0.86	0.28		
*E. siamensis*	15.38	11.02	0.00	
*P. oligactis*	16.63	12.00	1.64	0.00

## Data Availability

All sequences of the COI gene have been deposited under GenBank accession numbers PQ193904-PQ193914.

## References

[1] Nelson J.S., Grande T.C., Wilson M.V.H. (2016). Fishes of the World.

[2] Nelson J.S. (2006). Fishes of the World.

[3] Thacker C.E. (2013). Phylogenetic placement of the European sand gobies in Gobionellidae and characterization of gobionellid lineages (Gobiiformes: Gobioidei). Zootaxa.

[4] Chen I.S., Kottelat M., Miller P.J. (1999). Freshwater gobies of the genus *Rhinogobius* from the Mekong basin in Thailand and Laos, with descriptions of three new species. Zool. Stud..

[5] Chen I.S., Miller P.J. (2013). A new freshwater goby of *Rhinogobius* (Teleostei: Gobiidae) from Hainan Island, southern China. J. Mar. Sci. Technol..

[6] Kottelat M. (1989). Zoogeography of the fishes from Indochinese inland waters with an annotated check-list. Bull. Zool. Mus. Univ. Amst..

[7] McCraney W.T., Thacker C.E., Alfaro M.E. (2020). Supermatrix phylogeny resolves goby lineages and reveals unstable root of Gobiaria. Mol. Phylogenet. Evol..

[8] Thacker C.E., Roje D.M. (2011). Phylogeny of Gobiidae and identification of gobiid lineages. Syst. Biodivers..

[9] Xia J.H., Wu H.L., Li C.H., Wu Y.Q., Liu S.H. (2018). A new species of *Rhinogobius* (Pisces: Gobiidae), with analyses of its DNA barcode. Zootaxa.

[10] Chen I.S., Wang S.C., Shao K.T. (2022). A new freshwater gobiid species of *Rhinogobius* Gill, 1859 (Teleostei: Gobiidae) from northern Taiwan. Zootaxa.

[11] Suzuki T., Oseko N., Yamasaki Y.Y., Kimura S., Shibukawa K. (2022). A new species with two new subspecies of *Rhinogobius* (Teleostei: Gobiidae) from Yaeyama Group, the Ryukyu Islands, Japan. Bull. Kanagawa Pref. Mus. (Nat. Sci.).

[12] Maeda K., Kobayashi H., Iida M., Tran H.D. (2024). Taxonomy of freshwater gobies of the genus *Rhinogobius* (Oxudercidae, Gobiiformes) from central Vietnam, with descriptions of two new species. Zootaxa.

[13] Panitvong N. (2020). Freshwater Fishes of Thailand.

[14] Suvarnaraksha A., Utsugi K. (2023). A Field Guild of the Northern Thai Fishes.

[15] Hebert P.D.N., Cywinska A., Ball S.L., deWaard J.R. (2003). Biological identifications through DNA barcodes. Proc. R. Soc. Lond. B.

[16] Ahmed M.S., Datta S.K., Zhilik A.A. (2020). Molecular diversity of freshwater fishes of Bangladesh assessed by DNA barcoding. Bangladesh J. Zool..

[17] Ahmed M.S., Datta S.K., Saha T., Hossain Z. (2021). Molecular characterization of marine and coastal fishes of Bangladesh through DNA barcodes. Ecol. Evol..

[18] Bingpeng X., Heshan L., Zhilan Z., Chunguang W., Yanguo W., Jianjun W. (2018). DNA barcoding for identification of fish species in the Taiwan Strait. PLoS ONE.

[19] Seetapan K., Panprommin N., Wangkahart E., Ruenkoed S., Panprommin D. (2024). COI-high resolution melting analysis for discrimination of four fish species in the family Notopteridae in Thailand. Zool. Anz..

[20] Ward R.D., Zemlak T.S., Innes B.H., Last P.R., Hebert P.D.N. (2005). DNA barcoding Australia’s fish species. Philos. Trans. R. Soc. B.

[21] Panprommin D., Manosri R. (2022). DNA barcoding as an approach for species traceability and labeling accuracy of fish fillet products in Thailand. Food Control.

[22] Breitbart M., Kerr M., Schram M.J., Williams I., Koziol G., Peebles E., Stallings C.D. (2023). Evaluation of DNA metabarcoding for identifying fish eggs: A case study on the West Florida Shelf. PeerJ.

[23] Chen W., Li C., Li X., Li J., Li Y. (2022). Unraveling the drifting larval fish community in a large spawning ground in the Middle Pearl River using DNA barcoding. Animals.

[24] Nagao Natural Environment Foundation (2021). Fishes of the Indochinese Mekong.

[25] Sievers F., Higgins D.G. (2014). Clustal Omega, accurate alignment of very large numbers of sequences. Methods Mol. Biol..

[26] Altschul S.F., Gish W., Miller W., Myers E.W., Lipman D.J. (1990). Basic local alignment search tool. J. Mol. Biol..

[27] Tamura K., Stecher G., Kumar S. (2021). MEGA11: Molecular evolutionary genetics analysis version 11. Mol. Biol. Evol..

[28] Kimura M. (1980). A simple method for estimating evolutionary rates of base substitutions through comparative studies of nucleotide sequences. J. Mol. Evol..

[29] Saitou N., Nei M. (1987). The neighbor-joining method: A new method for reconstructing phylogenetic trees. Mol. Biol. Evol..

[30] Chen I.S., Cheng Y.H., Shao K.T. (2008). A new species of *Rhinogobius* (Teleostei: Gobiidae) from the Julongjiang Basin in Fujian Province, China. Ichthyol. Res..

[31] Kottelat M. (2001). Fishes of Laos.

[32] Larson H.K. (2009). Review of the gobiid fish genera *Eugnathogobius* and *Pseudogobiopsis* (Gobioidei: Gobiidae: Gobionellinae), with descriptions of three new species. Raffles Bull. Zool..

[33] Tan H.H., Lim K.K.P. (2011). Rediscovery of the bigmouth stream goby, *Pseudogobiopsis oligactis* (Actinopterygii: Gobiiformes: Gobionellidae) in Singapore. Nat. Singap..

[34] Smith H.M. (1945). The freshwater fishes of Siam, or Thailand. Bull. U.S. Natl. Mus..

[35] Sajjad A., Jabeen F., Ali M., Zafar S. (2023). DNA barcoding and phylogenetics of *Wallago attu* using mitochondrial COI gene from the River Indus. J. King Saud Univ. Sci..

[36] Hebert P.D.N., Ratnasingham S., de Waard J.R. (2003). Barcoding animal life: Cytochrome c oxidase subunit 1 divergences among closely related species. Proc. R. Soc. Lond. B.

[37] Shen Y., Guan L., Wang D., Gan X. (2016). DNA barcoding and evaluation of genetic diversity in Cyprinidae fish in the midstream of the Yangtze River. Ecol. Evol..

[38] Hebert P.D.N., Stoeckle M.Y., Zemlak T.S., Francis C.M. (2004). Identification of birds through DNA barcodes. PLoS Biol..

[39] Agorreta A., San Mauro D., Schliewen U., Van Tassell J.L., Kovačić M., Zardoya R., Rüber L. (2013). Molecular phylogenetics of Gobioidei and phylogenetic placement of European gobies. Mol. Phylogenet. Evol..

[40] International Union for Conservation of Nature (2023). The IUCN Red List of Threatened Species. https://www.iucnredlist.org/.

[41] Pornsopin P., Sirisuksa T., Kantiyawong S., Surajit T. (2022). Study on Cultivation of Chiangmai Stream Goby (Rhinogobius Chiengmaiensis Fowler, 1934).

